# Polymeric Microfiltration Membranes Modification by Supercritical Solvent Impregnation—Potential Application in Open Surgical Wound Ventilation

**DOI:** 10.3390/molecules26154572

**Published:** 2021-07-28

**Authors:** Mariusz Nowak, Dusan Misic, Anna Trusek, Irena Zizovic

**Affiliations:** 1 Faculty of Chemistry, Wroclaw University of Science and Technology, Wybrzeze Wyspianskiego 27, 50-370 Wroclaw, Poland; mariusz.nowak@pwr.edu.pl (M.N.); anna.trusek@pwr.edu.pl (A.T.); 2 Faculty of Biotechnology and Food Science, Wroclaw University of Environmental and Life Sciences, 51-651 Wroclaw, Poland; dusan.misic@upwr.edu.pl

**Keywords:** supercritical impregnation, microfiltration membranes, carvacrol, polyamide, carbon dioxide, insufflation, open surgical wound

## Abstract

This study investigated supercritical solvent impregnation of polyamide microfiltration membranes with carvacrol and the potential application of the modified membranes in ventilation of open surgical wounds. The impregnation process was conducted in batch mode at a temperature of 40 °C under pressures of 10, 15, and 20 MPa for contact times from 1 to 6 h. FTIR was applied to confirm the presence of carvacrol on the membrane surface. In the next step, the impact of the modification on the membrane structure was studied using scanning electron and ion beam microscopy and cross-filtration tests. Further, the release of carvacrol in carbon dioxide was determined, and finally, an open thoracic cavity model was applied to evaluate the efficiency of carvacrol-loaded membranes in contamination prevention. Carvacrol loadings of up to 43 wt.% were obtained under the selected operating conditions. The swelling effect was detectable. However, its impact on membrane functionality was minor. An average of 18.3 µg of carvacrol was released from membranes per liter of carbon dioxide for the flow of interest. Membranes with 30–34 wt.% carvacrol were efficient in the open thoracic cavity model applied, reducing the contamination levels by 27% compared to insufflation with standard membranes.

## 1. Introduction

Impregnation of solid matrices using high-pressure carbon dioxide has numerous advantages in comparison to conventional impregnation techniques as it overcomes significant drawbacks of the conventional methods, such as low diffusion rate, extensive effluent, solid waste generation, uneven impregnation, overdosing of the impregnate, long processing times, high energy demand, etc. [1]. Impregnation using supercritical carbon dioxide (scCO_2_) has been applied on an industrial scale in wood impregnation [2,3] and textile dyeing [4,5], enabling complete effluent elimination in these productions. Commercially competitive solutions for other processes have been proven as well [1]. In supercritical solvent impregnation (SSI), an active substance is dissolved in the supercritical fluid, which acts as a transport medium that is able to quickly penetrate the solid matrix due to the absence of surface tension. Consequently, SSI offers a unique advantage of solid impregnation throughout the whole volume. Another benefit of SSI is the possibility of impregnation of finished polymeric forms, as demonstrated with hip and knee endoprosthesis [6,7] and contact lenses [8,9]. An extensive review on the application of SSI in polymeric implant loading with drugs was provided by Champeau et al. [10]. The authors pointed out that the advantages of SSI included the possibility of operating under mild temperatures, processing thermosensitive active pharmaceutical ingredients (APIs), and obtaining a final impregnated matrix that is free of any solvent residue. The effects of the processing conditions and the presence of a cosolvent on drug loading were analyzed. The successful utilization of SSI in the fabrication of drug-loaded ophthalmic devices, stents, oral drug delivery systems, and scaffolds for cell tissue engineering was highlighted [10]. Another important application of SSI is designing active food packaging and carrier materials loaded with nutraceutical compounds [11]. In a review, Rojas et al. [11] thoroughly discussed the effects of pressure, temperature, decompression rate, and contact time on active substance loading in polymers aimed for food packaging. The authors highlighted that SSI allows incorporation of active compounds with a high or low affinity towards a polymer. In the first case, this occurs through molecular dispersion based on the strong interactions between the active compound and the polymer, while in the second case, it occurs through the deposition of the active component in the swollen polymer due to fast decompression. The review also discussed the utilization of aerogel as nutraceutical carriers and presented published studies on aerogel SSI with active components [11].

Our recent results [12,13] showed the feasibility of commercial microfiltration membranes impregnation with thymol via SSI to add strong antibacterial properties to these defined polymeric structures. The results indicated thymol dispersion at the molecular level in the polymer matrix of polyamide [12] and cellulose acetate membranes [13] with their microstructure preserved. This study further explores the SSI of commercial polyamide membranes with carvacrol and the potential application of these defined structures loaded with antibacterial substance in open surgical wound ventilation. Unlike thymol, carvacrol is in a liquid state under atmospheric conditions. This is advantageous for its application in materials design using supercritical fluids as there is no possibility of equipment clogging due to crystal formation.

Insufflation of carbon dioxide in the thoracic cavity during open-heart surgery has been used to prevent arterial air metabolism since the 1950s [14]. Scientists, surgeons, and clinical engineers have made efforts to better understand the de-airing process and develop convenient devices ever since [14,15,16,17,18]. It has also been shown that carbon dioxide insufflation during open thoracic cavity surgeries might be used to reduce exogenous perioperative tissue contamination and occurrence of surgical site infections that are most commonly caused by *Staphylococcus aureus* [16,17,18]. A source of perioperative exogenous contamination can be an active person present in the operating room as each individual emits thousands of bacteria-carrying particles every minute [16]. In addition, laminar ultraclean airflow from the ceiling downwards helps convey airborne particles from the surgical team to the operating table and increases direct airborne contamination of the open surgical wound [16]. Persson and van der Linden [16] showed that intraoperative surgical wound ventilation with carbon dioxide using a proposed gas diffuser might prevent air embolism and significantly reduce the risk of tissue contamination at the same time. Persson et al. [17] further proposed antiseptic open surgical wound ventilation using carbon dioxide carrying gasified ethanol, demonstrating its efficiency in contamination prevention. Baumann and Cater [18] also showed that insufflation with warm carbon dioxide significantly reduced open wound contamination compared to the procedure without insufflation.

In this study, we explored antiseptic de-airing in an open thoracic cavity model using membranes impregnated with an antibacterial substance by SSI. Microfiltration membranes are an unavoidable part of every device proposed for open surgical wound ventilation as they aim to remove bacteria from the carbon dioxide stream before insufflation. The main idea of this study was to investigate whether membranes impregnated by SSI could serve as a bacterial filter and a source of antiseptic substance at the same time. Carvacrol was selected as an antibacterial compound because of its GRAS (generally recognized as safe) status [19]. In addition, carvacrol possesses strong antibacterial properties [20], excellent solubility in carbon dioxide [21] and has previously been applied in SSI [22,23,24]. This study evaluated the impregnation yield of carvacrol in commercial polyamide membranes by laboratory scale SSI at 40 °C under pressures of 10, 15, and 20 MPa for different times. FTIR analysis was used to confirm the presence of carvacrol on the polymer surface. The effect of the impregnation process on the microstructure of the membranes was examined by combined scanning electron and ion beam microscopy and cross-filtration tests. Subsequently, the release of carvacrol in the carbon dioxide flowing through the membrane was determined. Finally, an open thoracic cavity model was applied to compare contamination rates in insufflation using a neat polyamide membrane as a bacterial filter for carbon dioxide and insufflation, with a carvacrol-loaded polyamide membrane used as a bacterial filter and a source of antibacterial compound at the same time.

## 2. Materials and Methods

### 2.1. Materials

Commercial polyamide microfiltration membranes with an average pore diameter of 0.2 µm and a membrane diameter of 47 mm were purchased from GE Healthcare WhatmanTM, Germany (Cat. No. WHA10414012). Carvacrol (purity ≥98%) was supplied by Sigma-Aldrich, Germany. Carbon dioxide (purity >99.99%) was provided by Air Liquid, Wroclaw, Poland.

### 2.2. Methods

#### 2.2.1. Supercritical Solvent Impregnation (SSI)

Polyamide membranes were impregnated with carvacrol in a 280 mL high-pressure vessel (Eurotechnica GmbH, Bargteheide, Germany) equipped with a heating jacket. Water was used as a heating fluid, and its circulation was provided by a heating bath circulator (Jeio Tech Co., Ltd., Daejeon, Korea). Around 2 mL of carvacrol was placed in a glass container in the middle of the vessel. Two membranes were placed on the left of the carvacrol, and two were placed on the right. The mass of each membrane was approximately 0.07 g. After the desired temperature was reached, carbon dioxide was pumped into the system by an air-driven gas booster (Eurotechnica GmbH, Barghteheide, Germany) until the working pressure was obtained. At the end of each run, the system was decompressed with an intermediate decompression rate of 0.25 MPa/min. The experiments were performed at a temperature of 40 °C and pressures of 10, 15, and 20 MPa. The impregnation time was varied from 1 to 6 h. The selection of temperature and decompression rate was based on preliminary experiments, where a temperature of 50 °C yielded slower impregnation kinetics. Slow decompression rate (0.15 MPa) did not considerably affect the impregnation yield, and fast decompression was not considered because of possible adverse effects on the membrane microstructure. Carvacrol loading (impregnation yield) was determined gravimetrically and calculated according to the following equation:

(1) $$I=\frac{m_{carv}}{m_{im}}\cdot 100\%$$

where *m_carv_* is the mass of impregnated carvacrol obtained as a mass difference between the impregnated and neat membrane, and *m_im_* is the mass of the impregnated membrane. An analytical balance with an accuracy of 0.01 mg was used for the measurements. Experiments were performed in triplicates, and the standard deviation was calculated according to the following formula:

(2) $$SD=\sqrt{\frac{\sum {\left(Y_{i}-Y_{av}\right)}^{2}}{N}}$$

where *Y_i_* is the variable value (carvacrol loading) obtained in experiment *i*, *Y_av_* is the average value of the variable, and *N* is the number of experiments.

#### 2.2.2. FTIR Analyses

The presence of carvacrol on the surface of membranes was confirmed by Fourier transform infrared (FTIR) spectroscopy. The spectra of impregnated carvacrol and neat polyamide membranes were recorded in ATR mode using a Nicolet iS50 spectrometer (Thermo Fisher SCIENTIFIC, Waltham, MA, USA) with a resolution of 4 cm^−1^ at wavenumbers in the range of 500–4000 cm^−1^.

#### 2.2.3. Microscopy

A two-beam microscope SEM/Ga-FIB FEI Helios NanoLab™ 600i (FEI, Thermo Fisher Scientific, Eindhoven, the Netherlands) was used to investigate the structural properties of neat and impregnated membranes. The microscope comprises ultrahigh resolution electron and ion microscopy. The energy-focused beam of gallium ions allows the selective removal of the preparation material and modification at the nanoscale, such as the sample cross sections. Before the analyses, the samples were coated with gold.

#### 2.2.4. Tests in a Cross-Filtration Unit

**Figure A1:**
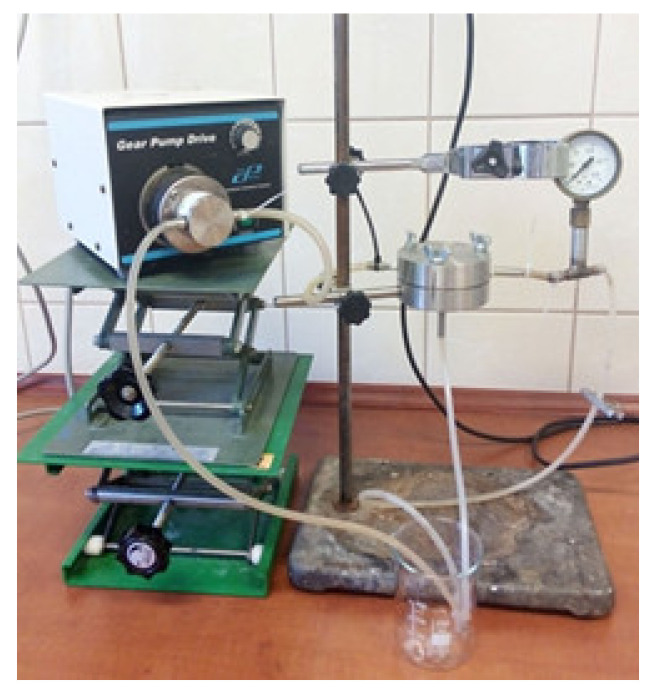
The laboratory filtration unit: Gear pump (Cole Parmer, Vernon Hills, IL, USA), steel module for membrane, and monometer. Pressure is generated by throttling.

The impact of swelling on the membrane structure and the resulting solvent (water) permeability was investigated by comparing the water permeate stream for neat and impregnated membranes in a cross-flow system. The water permeate stream flow rate was measured for different values of transmembrane pressure. A cross-flow laboratory system, shown in Figure A1 (Appendix A), was used for these measurements. The transmembrane pressure was varied in the range of 5–20 kPa.

#### 2.2.5. Release of Carvacrol in Carbon Dioxide

The same filtration stand employed in the cross-filtration tests (steel module, Figure A1) was used to measure the release of carvacrol from the membranes into carbon dioxide. The stand was connected to the 280 mL high-pressure vessel (Eurotechnica GmbH, Bargteheide). A rotameter was placed after the membrane. After the high-pressure vessel was filled with carbon dioxide to the pressure of 2.5 MPa, the flow through the system commenced. The flow rate was regulated by metering valves (one placed between the CO_2_ cylinder and the vessel and the other between the vessel and the filtration stand). A flow rate of 5 L/min was used for this study. The release was followed gravimetrically by measuring the mass of the membrane in predetermined time intervals.

#### 2.2.6. Thoracic Cavity Model Protocol

**Figure 1:**
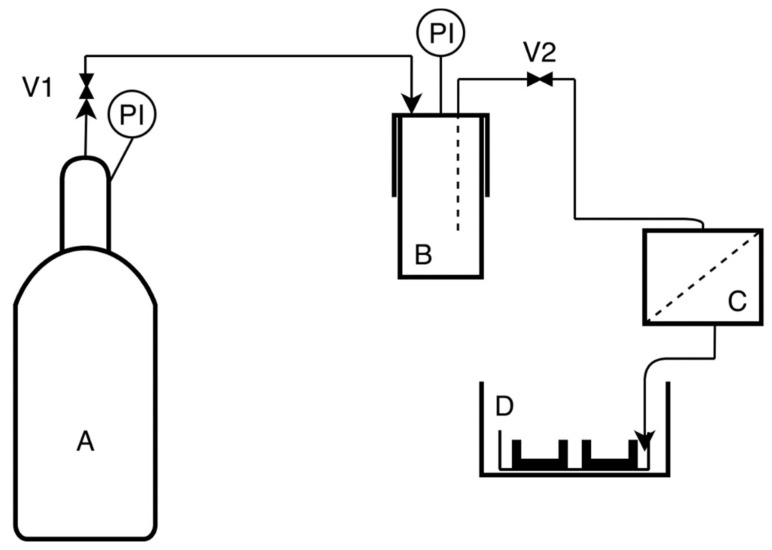
The open thoracic cavity model setup; (**A**): CO_2_ cylinder, (**B**): high-pressure vessel (CO_2_ pressure approximately 2.5 MPa), (**C**): membrane stand, (**D**): table with the model containing blood agar plates.

The study protocol was similar to the one used by Persson and Linden [16]. The experiments were performed in a laboratory with closed windows where one person worked in a usual regime without leaning over the model. The airborne bacteria were collected in two 9 cm standard blood agar plates as a nutrient medium. The schematic presentation of the experimental setup is given in Figure 1. The open thoracic cavity model that contained nutrient medium plates was oval shaped with length, width, and height of 23, 15, and 6 cm, respectively. The model was placed at the table lifted 50 cm from the ground in an enclosure with length, width, and height of 35, 28, and 22 cm, respectively, to prevent staff from approaching the model. The open-ended tube was pointed towards the center of the model [16]. The model, membrane stand, and all piping were sterilized with ethanol before each experiment. Every experiment started by allowing gas to flow into the model for two minutes before the agar plates were opened by a person wearing sterile gloves. After one hour of gas flow at the rate of 5 L/min, the agar plates were closed while the gas was still flowing and sent for analysis. The experiments were performed in five replicates with neat polyamide membranes and membranes with around 30 wt.% carvacrol. The experiments were also performed without carbon dioxide insufflation for contamination comparison.

#### 2.2.7. Microbial Analyses

Investigation of the antibacterial effect of insufflation was performed as described in Section 2.2.6. For this purpose, 5% Columbia sheep blood agar in Petri dishes fi 90 (Becton Dickinson, Heidelberg, Germany) was used. The general guidelines we used in microbiological testing can be found in EN-ISO 7218:2007/A1:2013 “Microbiology of food and feeding stuffs. General requirements and guidance for microbiological examinations”. After the experiments, plates were immediately inserted into a thermostat and incubated at 37 °C for 72 h under aerobic conditions, after which the grown colonies were counted.

## 3. Results and Discussion

### 3.1. Supercritical Solvent Impregnation

**Figure 2:**
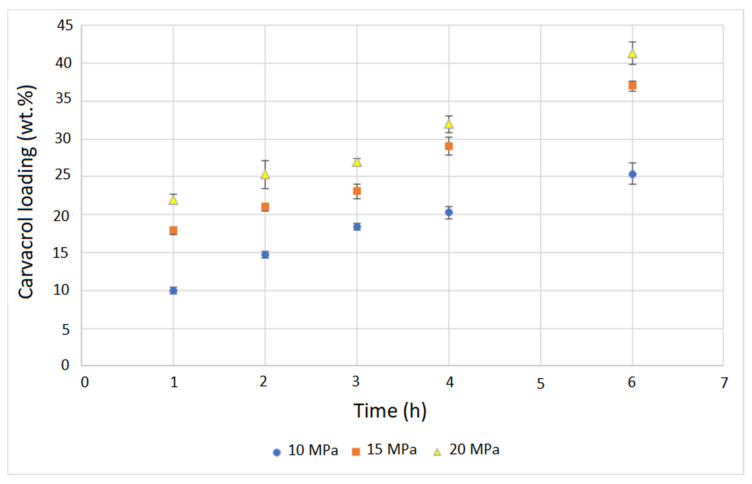
Kinetics of polyamide membranes SSI with carvacrol.

The SSI experiments were carried out at a temperature of 40 °C under pressures of 10, 15, and 20 MPa for contact times of 1, 2, 3, 4, and 6 h. Experiments were performed in triplicates, and four membranes were impregnated during each run. The mean values and standard deviations of the obtained carvacrol loadings are presented in Figure 2. The loadings were determined gravimetrically, as described in Section 2.2.1. It is worth noting that there are two characteristics of this experimental approach that may affect the measurement accuracy. The first is that SSI is a two-way mass transfer process that includes polymer impregnation and extractable compound removal from the polymer. The second is the time needed for CO_2_ desorption. We determined the fraction of extractables in the membranes to be approximately 0.3 wt.%. This would mean our loading values were underestimated by the same amount. However, we observed that CO_2_ desorption from the membranes was a slow process and that several days were needed for complete desorption. Measurement of samples half an hour after decompression led to measured value overestimation of around 0.44 wt.%. Finally, our values were overestimated by approximately 0.14%, which is not a significant error for high loadings and lies in the domain of experimental error (Figure 2).

**Figure A2:**
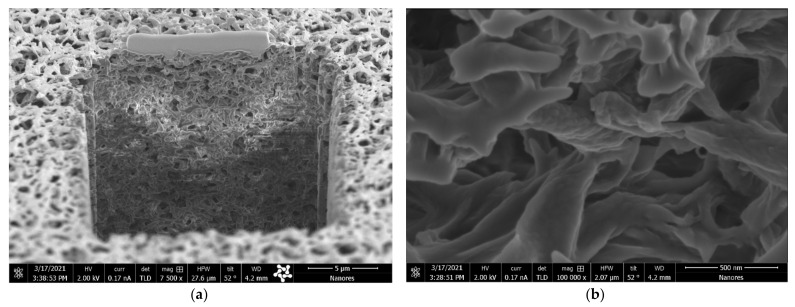
The cross section of a polyamide membrane exposed to pure scCO_2_ at 15 MPa and 40 °C for 3 h (bar = 5 µm (**a**), bar = 500 nm (**b**)). The bump-like structure of the virgin polymer is visible in the micrograph (**b**). Unpublished material from the study [12].

**Figure A3:**
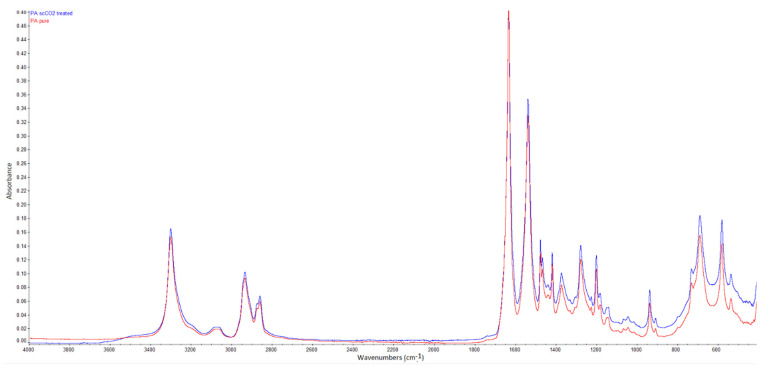
FTIR spectra of a neat and scCO_2_-treated membrane at 20 MPa and 40 °C for 3 h.

The results presented in Figure 2 indicate very fast impregnation at pressures of 15 and 20 MPa with carvacrol loadings of 17.9% and 22%, respectively, after just one hour. The larger the pressure, the faster was the impregnation. This behavior indicates that carvacrol solubility in scCO_2_ had the dominant effect on the rate of the process. Specifically, the molar fraction of carvacrol in scCO_2_ increased from 0.014 to 0.034 and 0.051 when the pressure increased from 10 to 15 and 20 MPa [21], thereby allowing faster impregnation at higher pressures. It is important to stress that carvacrol was present in excess in the system, allowing for the maintenance of scCO_2_ saturation during impregnation. This SSI mechanism necessary to obtain high loadings has been explained by Rojas et al. [11]. Another important phenomenon that occurs during SSI is polymer swelling due to exposure to scCO_2_, which enables fast impregnation. The swelling is proportional to the sorbed amount of carbon dioxide [25], and for many tested semicrystalline polymers, it is positively affected by the pressure [26]. A recent study [27] revealed the increase of scCO_2_ permeation through glassy polyamide membranes with a pressure increase from 8 to 12 MPa at temperatures of 35–55 °C. Therefore, we can assume that the sorption and swelling effects also contributed to faster impregnation under higher pressure. However, sorption measurements are needed to better understand this impact_._ Our previous study [12] using SEM and ion beam microscopy demonstrated that polymer swelling due to exposure to pure scCO_2_ (without impregnation) was temporary. The polymer returned to the last state after the exposure and retained a bumped-like structure of the virgin polymer (Figure A2, Appendix A). The scCO_2_-treated membrane behaved in the same manner in cross-flow filtration tests as well [12]. Here, we also provide FTIR spectra of the neat and scCO_2_-treated polymer (Figure A3, Appendix A), where no difference was noticed.

**Figure A4:**
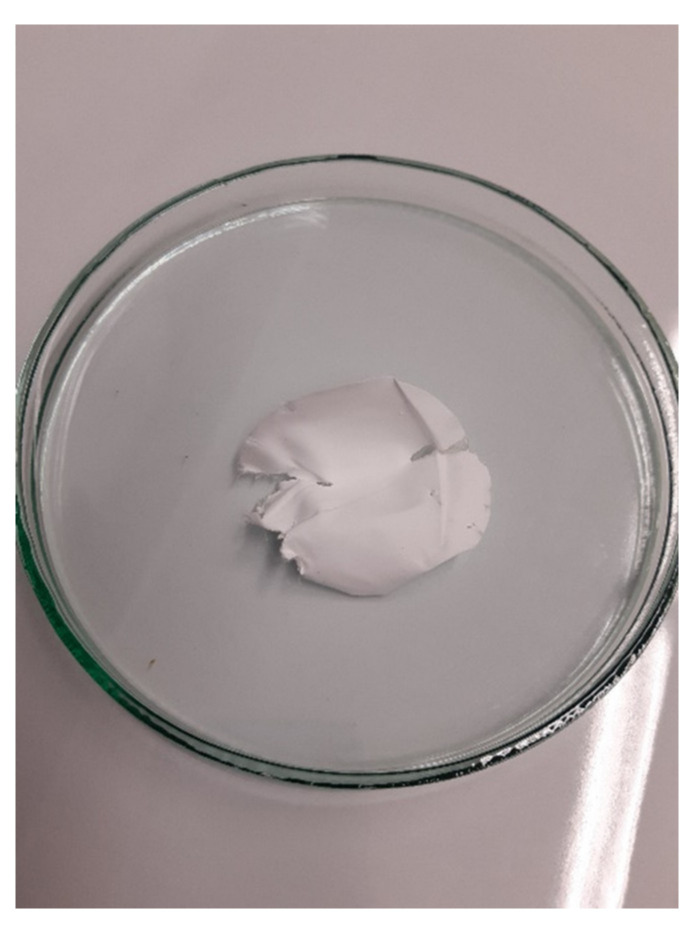
The appearance of the polyamide membrane with 54% carvacrol. The membrane was purposely bent during the SSI and could not be unfolded without damage.

Similar impregnation kinetics and pressure impact were reported in the SSI of cellulose acetate [23] and cotton gauze [22] with carvacrol. High carvacrol loadings of up to 60% were reported for cellulose acetate in the form of beads [23] and around 30% for cellulose acetate films [24]. In the case of cotton gauze, carvacrol loadings of up to 14% were attainable [22]. Our previous study [12] on SSI of polyamide microfiltration membranes with thymol reported loadings of up to 43% and fast impregnation in the first hour of the process. SSI of polyamide nanofibers with thymol was also previously investigated [28], and thymol loadings of up to 59.2% were reported. After six hours of SSI in our study, average carvacrol loadings of 25.4%, 37%, and 41.3% were obtained at 10, 15, and 20 MPa, respectively. Thus, even higher loadings were attainable in this process. With longer impregnation times at 20 MPa, a maximal carvacrol loading of 54% was reached. However, the swelling effect caused membrane damage (Figure A4, Appendix A).

**Figure 3:**
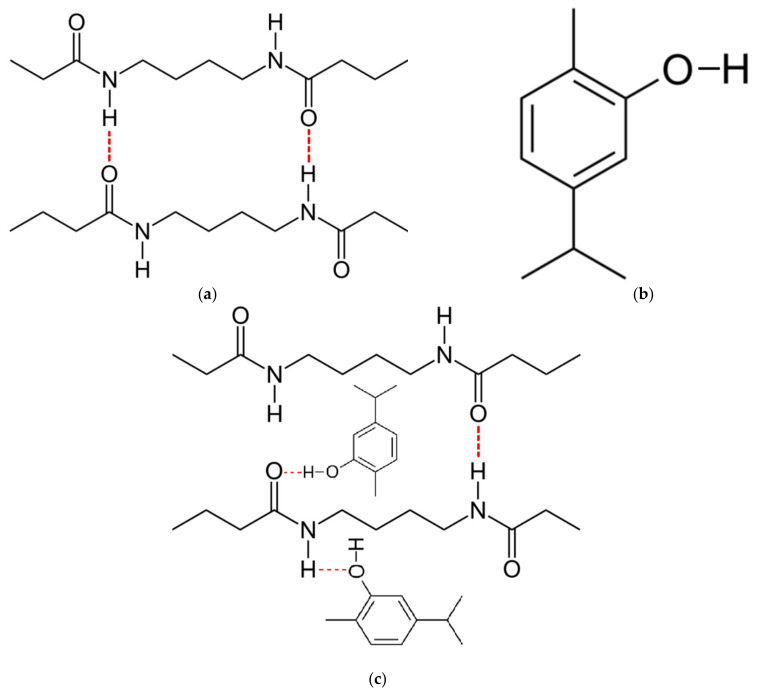
Chemical formulas of polyamide with hydrogen bonds between polymer chains shown in red (**a**) and carvacrol (**b**); possible hydrogen bonding in carvacrol-impregnated polyamide (**c**).

High loading of an active compound in a polymer is possible if strong interactions exist between the compound and functional groups of the polymer [11,28]. The chemical formulas of polyamide (Figure 3a) and carvacrol (Figure 3b) reveal the possibility for hydrogen bonding between the carbonyl group of polyamide and hydrogen from the hydroxyl group of carvacrol as well as hydrogen from the imino group of polyamide and oxygen from the hydroxyl group of carvacrol (Figure 3c). It is also important to stress that hydrogen bonding exists between carbonyl and imino groups in polyamide (colored red in Figure 3a).

### 3.2. FTIR Analyses

**Figure 4:**
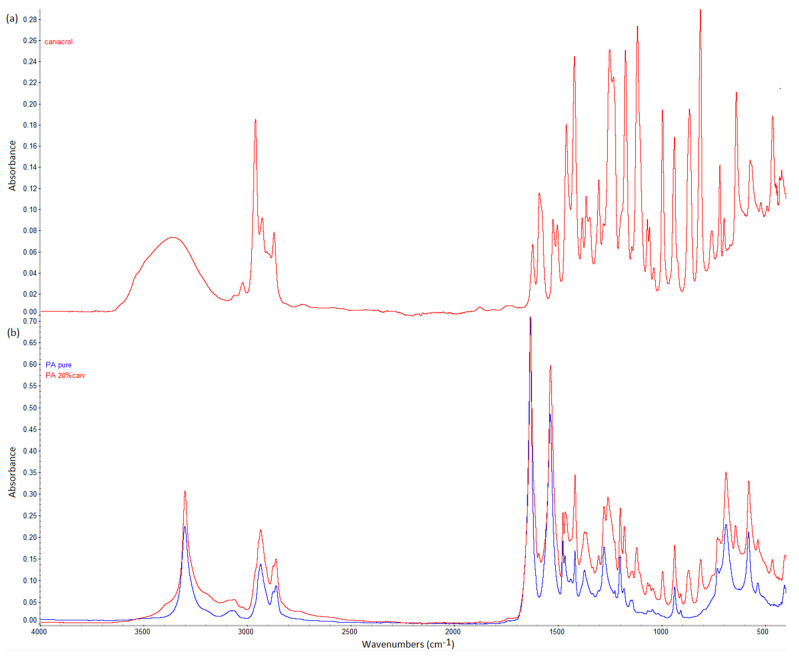
FTIR spectra of carvacrol (**a**) and neat and impregnated polyamide membranes (**b**).

FTIR spectra of carvacrol, a neat polyamide membrane, and a polyamide membrane with 20% carvacrol are presented in Figure 4. The wavenumbers of the characteristic bands are provided in Table 1. In the spectrum of carvacrol, a band belonging to the –OH stretching vibration was detected at 3378 cm^−^^1^, while bands attributed to aliphatic –CH_2_/–CH_3_ stretching were recorded at 2958, 2926, and 2868 cm^−1^ [29,30,31]. The band at 811 cm^−1^ could be assigned to C–H out-of-plane wagging [29,30,31]. Peaks related to carvacrol ortho and 1:2:4 substitution were visible at 1115 and 994 cm^−1^, respectively [29,30,31]. The key characteristic peaks of carvacrol [29] were detected as well and are presented in Table 1.

The characteristic bands for polyamide could be seen in the spectrum of the neat polyamide membrane. The bands at 3299 and 3076 cm^−1^ originated from N–H stretching vibration [32,33]. The bands at 2933 and 2859 cm^−1^ were assigned to CH_2_ asymmetric and symmetric stretching vibrations. The band at 1631 cm^−1^ was attributed to the C=O stretching of amide, while the band at 1536 cm^−1^ corresponded to the N–H bending of amide [32,33,34]. The band at 689 cm^−1^ was assigned to the bending of the O=C–N group [34]. New bands appeared in the spectrum of the impregnated membrane in the region between 800 and 1200 cm^−1^, which is the fingerprint region for aromatic compounds [31,35]. The band at 810 cm^−1^ was assigned to C–H out-of-plane wagging [29,31]. The band at 865–870 cm^−1^ was the key characteristic peak of carvacrol [30,31]. The band at 995 cm^−1^ and peak at 1114–1121 cm^−1^ corresponded to 1:2:4 substitution and ortho substitution of carvacrol, respectively [29,30,31]. The characteristic bands of both spectra are presented in Table 1.

**Figure 5:**
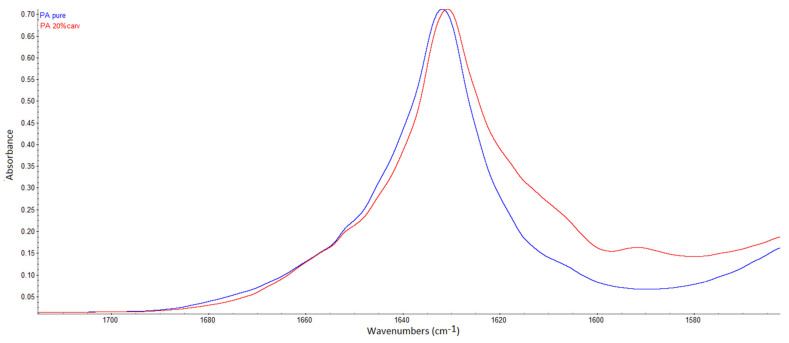
C=O stretching vibration band in polyamide (peak at 1631 cm^−1^) and a slightly broader band in the impregnated sample (peak at 1630 cm^−1^).

**Figure 6:**
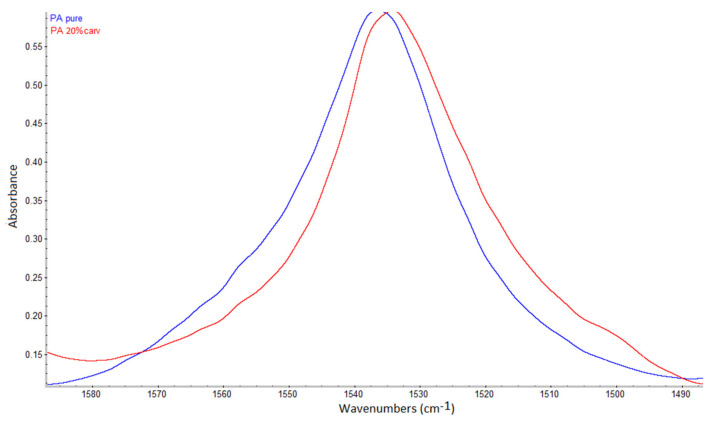
N–H bending vibration band in polyamide (peak at 1536 cm^−1^) and carvacrol-impregnated polyamide (peak at 1534 cm^−1^).

**Figure 7:**
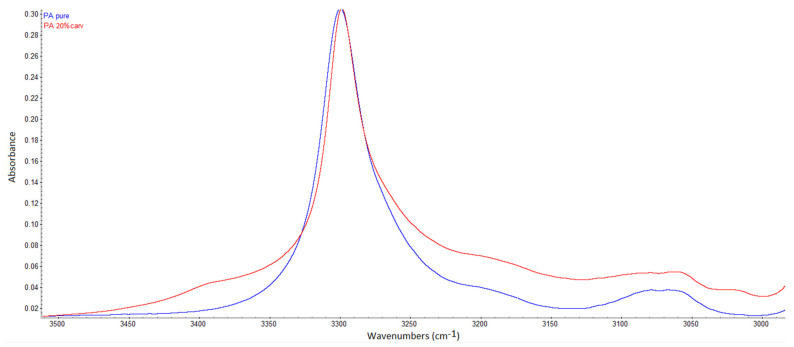
N–H stretching vibration bands in polyamide (peaks 3299 and 3076 cm^−1^) and carvacrol-impregnated polyamide (3298 and 3065 cm^−1^).

**Figure A5:**
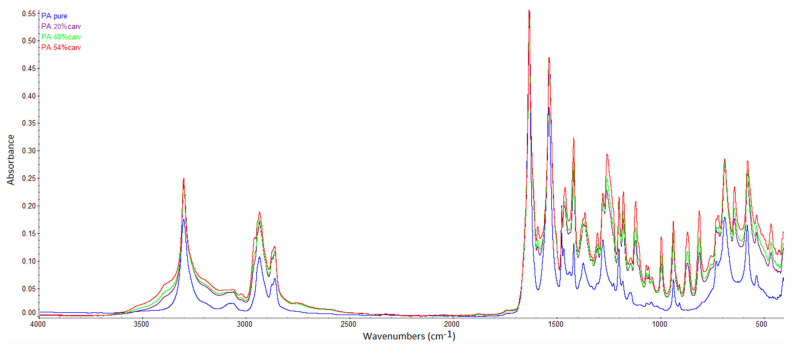
Spectra of neat and carvacrol-impregnated polyamide membranes.

**Figure A6:**
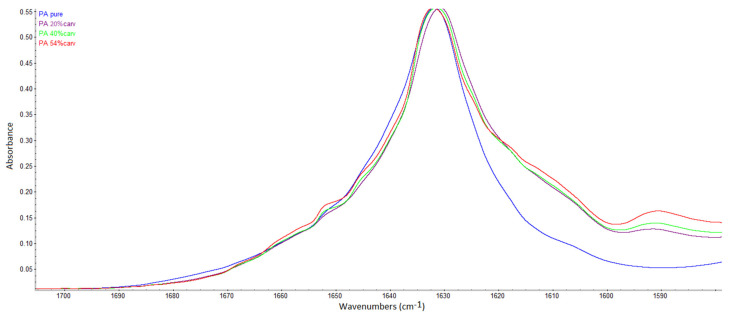
C=O stretching vibration band in neat and carvacrol-impregnated polyamide membranes.

**Figure A7:**
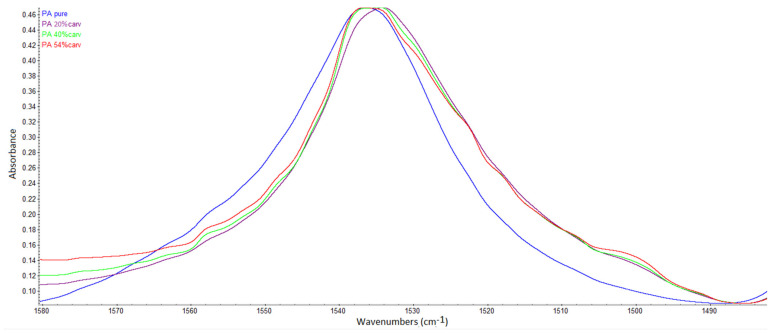
N–H bending vibration band in neat and carvacrol-impregnated polyamide membranes.

**Figure A8:**
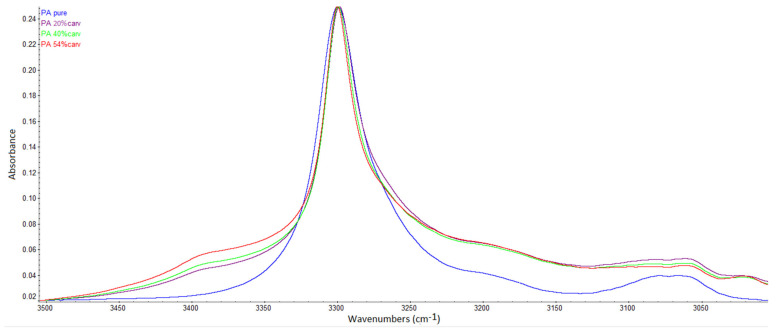
N–H stretching vibration bands in neat and carvacrol-impregnated polyamide membranes.

In FTIR spectra, the presence of hydrogen bonding may be detected as a shift of the functional group’s band towards a lower wavenumber, possibly accompanied by a broader band appearance [36,37,38]. Kaziran and Martirosyan [36] demonstrated the interaction (hydrogen bond) between the carbonyl group of polyvinylpyrrolidone (PVP) and the hydroxyl group of ibuprofen using FTIR. The stretching C=O band of the virgin PVP was shifted towards a lower wavenumber (from 1682 to 1636 cm^−1^), which was attributed to hydrogen bonding. Unlike in PVP, in polyamide, strong hydrogen bonding exists between polymer chains (Figure 3a). During SSI, carvacrol interferes with the already existing bonding between polymer chains, making its own bonds at the same locations. Thus, the difference will be less visible in FTIR spectra. However, the consequence of this phenomenon is the possibility of high carvacrol loadings in polymer, attainable only when strong interactions exist between the polymer and the active substance [11]. The other proof of hydrogen bonding with carvacrol is the permanent swelling of the polymer, which will be discussed later. Analyzing the spectra, a closer look at the C=O stretching vibration (Figure 5), N–H bending vibration (Figure 6), and N–H stretching vibration (Figure 7) of the pure and impregnated polyamide revealed slightly shifted bands towards lower wavenumbers in the impregnated polymer. In the region of C=O stretching vibration (Figure 5), a broader C=O stretching band was detected in the impregnated sample, with a slight peak shift from 1631 to 1630 cm^−1^. In the same spectrum, an appearance of a new weak band at 1590 cm^−1^ was also noticed. The new band could be attributed to the aromatic C=C stretch [39] in carvacrol. The most considerable shift detected was for the N–H stretching band from 3076 to 3065 cm^−1^ (Figure 7). The observed shifts were not so substantial, as in PVP, because one kind of hydrogen bonding replaced another in impregnated polyamide. The effect was further analyzed for higher carvacrol loadings (40% and 54%), and similar results were obtained. The spectra are provided in Figure A5, Figure A6, Figure A7 and Figure A8 (Appendix A).

### 3.3. Modification Impact on Membrane Microstructure

Like its isomer thymol, carvacrol with its hydroxyl group can establish hydrogen bonds with functional groups of polymers. This phenomenon allows for high loadings of these substances into the polymers, such as cellulose acetate and polyamide [12,28,40,41]. However, hydrogen bonding with carvacrol weakens electrostatic interactions between the polymer chains themselves, inevitably leading to permanent polymer swelling. The question is whether this swelling will affect the microstructure and performance of the membranes. These effects were investigated using the combined scanning electron and ion beam microscopy and cross-filtration tests.

**Figure 8:**
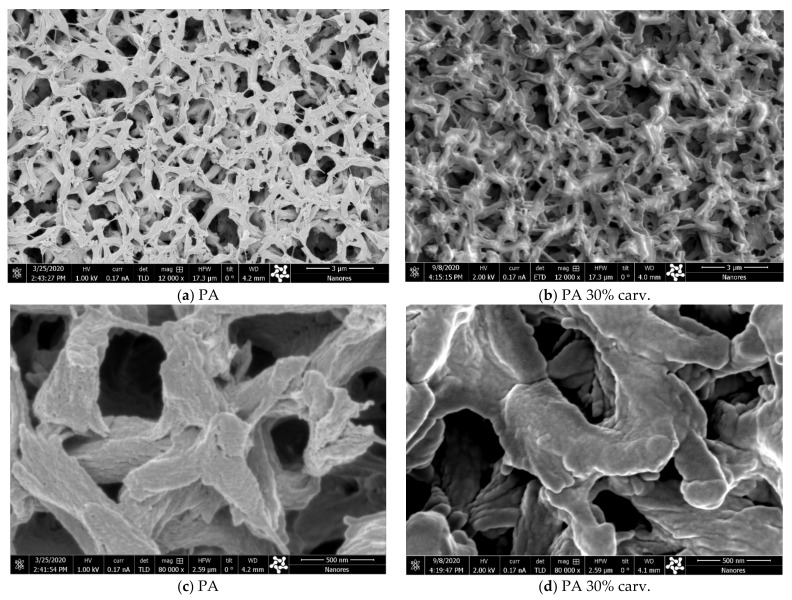
SEM images of surfaces of a neat polyamide membrane (PA) and polyamide membrane with 30 wt.% carvacrol (PA 30% carv.). Bar = 3 µm in micrographs (**a**,**b**); bar = 500 nm in micrographs (**c**,**d**).

In a previous study [12], the swelling effect was slightly visible in polyamide membranes with 20% thymol and clearly visible in samples with 35% loading. Here, we present scanning electron microscopy (SEM) images of the surfaces of neat and modified polyamide membranes with around 30 wt.% carvacrol (Figure 8). No change in membrane surface appearance could be spotted under magnification of 12,000× (Figure 8a,b). However, magnification of 80,000× (Figure 8c,d) revealed a less prominent bump-like structure in the sample with 30% carvacrol, indicating that polymer swelling had occurred.

**Figure 9:**
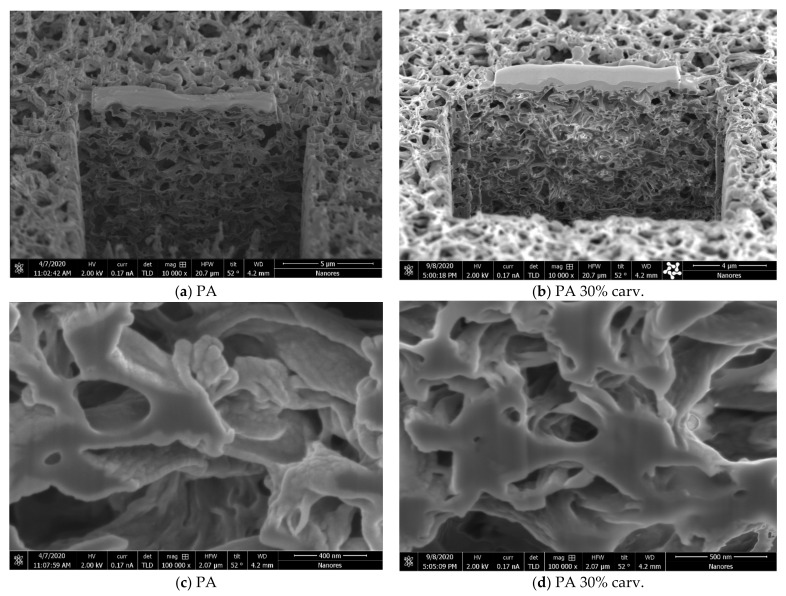
SEM images of cross sections of a neat polyamide membrane (PA) and polyamide membrane with 30 wt.% carvacrol (PA 30% carv.). In micrographs (**a**–**d**), bar values are 5 µm, 4 µm, 400 nm, and 500 nm, respectively.

**Figure A9:**
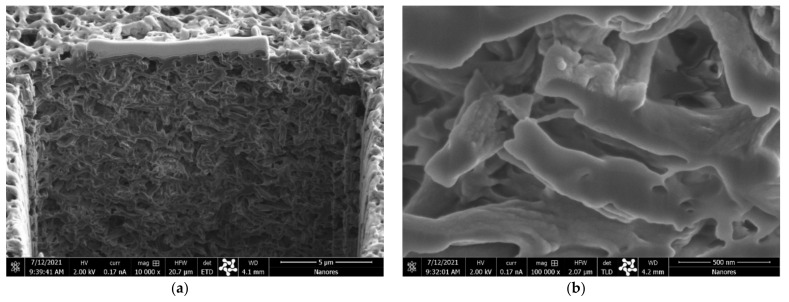
SEM images of the cross section of a polyamide membrane with 43% carvacrol (bar = 5 µm (**a**), bar = 500 nm (**b**)).

The combination of SEM and energy-focused beam of gallium ions allowed for sample modification (cross section) during SEM analyses. The SEM images of the cross sections of neat and modified membranes with 30 wt.% carvacrol are presented in Figure 9. No significant change in the membrane structure was visible under magnification of 10,000× (Figure 9a,b), indicating that the membrane performance shouldn’t be significantly affected by the modification. However, larger magnifications (100,000×) revealed the swelling effect and a less prominent bump-like polymer in the sample with 30% carvacrol. A similar phenomenon was observed in the membrane with the highest carvacrol content of 43% obtained under the investigated conditions (20 MPa, 40 °C, 3 h), as shown in Figure A9 (Appendix A).

**Figure 10:**
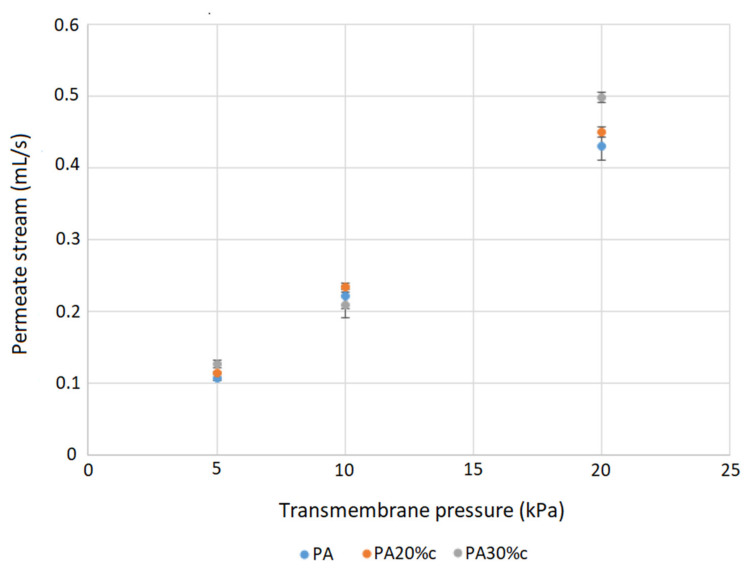
Permeate stream through a neat polyamide membrane (PA) and membranes with 20% (PA20%c) and 30% (PA30%c) carvacrol.

Tests in a cross-flow filtration system were performed to further investigate the impact of swelling on the membrane performance. The water permeate flow through the membrane was measured for different transmembrane pressures for a neat polyamide membrane and modified membranes with carvacrol content in the range 20–43 wt.%. The results showed similar behavior of the neat membrane and membranes with carvacrol content below 30%. However, membranes with carvacrol content in the range of 30–43% showed around 10% higher flow rate for the transmembrane pressure of 20 kPa. The data obtained for a neat polyamide membrane and membranes with characteristic carvacrol loadings of 20% and 30% are shown in Figure 10. In a previous study [12], comparable results were obtained, with polyamide membrane with 20% thymol retaining the neat membrane’s permeate flow rate. However, the membrane with 35% thymol showed a slight increase in the permeate stream. Based on the obtained results, it can be concluded that there was no significant change in the membrane microstructure and functionality due to the swelling effect.

It is important to stress that no differences could be noticed in membranes with the same loading of carvacrol produced under different SSI conditions by SEM and FTIR analyses and cross-filtration tests. This observation is in accordance with the findings of previous studies on polyamide [12,28] and cellulose acetate [40,41] SSI with thymol, where the same phenomenon of hydrogen bonding and high loadings exists. The studies confirmed that the physical properties, release kinetics, and biological activity of the final material depend only on the active substance loading and not on the SSI conditions.

### 3.4. Carvacrol Release in Carbon Dioxide

**Figure 11:**
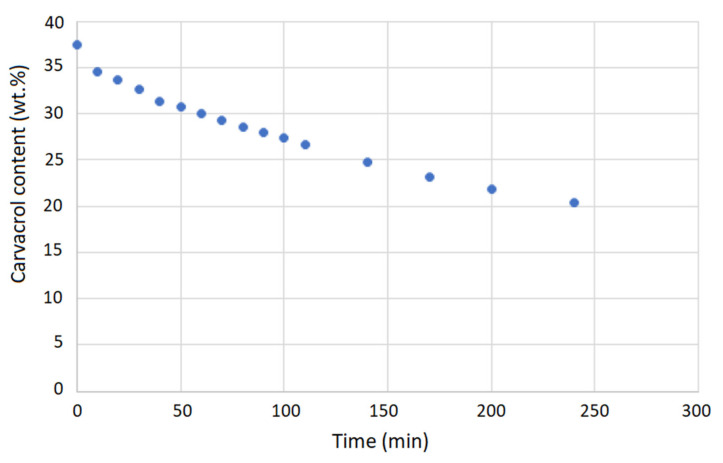
Carvacrol content in the membrane as a function of time (CO_2_ flow rate: 5 L/min, initial carvacrol content: 37%).

**Figure 12:**
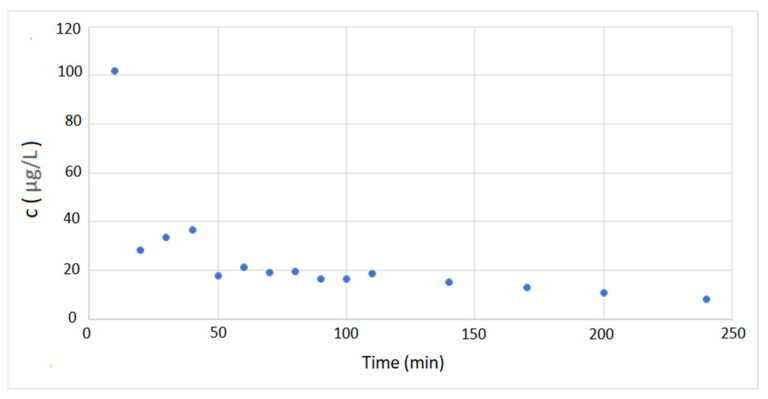
Carvacrol concentration in carbon dioxide as a function of time (CO_2_ flow rate: 5 L/min, initial carvacrol content: 37%).

**Figure A10:**
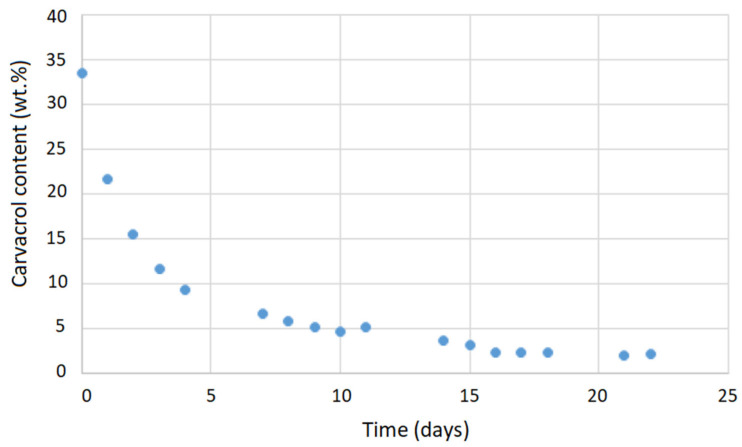
Carvacrol release in air at 25 °C.

The first step in evaluating the potential application of carvacrol-loaded membranes in open surgical wound ventilation is to investigate the release of carvacrol in carbon dioxide under the flow used for wound “de-airing”. According to the literature, the most often applied flow rates in open surgical wound ventilation in Japan range from 2 to 5 L/min [15]. Other studies have indicated flow rates of interest as 2–10 L/min [14], 5 and 10 L/min [16], 5 L/min [17], and 10 L/min [18]. We selected a 5 L/min flow rate of carbon dioxide through the membrane for our experiments. A cross-flow membrane stand was connected to a high-pressure vessel with carbon dioxide in which the pressure of 2.5 MPa was maintained. Metering valves regulated the flow, and a rotameter was placed behind the membrane. The carvacrol release results are presented in Figure 11. The release trend was almost linear after the first 10 min, with an average decrease in carvacrol loading of around 5% per hour of CO_2_ flow or around 5.5 mg of carvacrol per hour. The corresponding average concentration of carvacrol in carbon dioxide would be 18.3 µg/L. The concentration of carvacrol in carbon dioxide as a function of time is shown in Figure 12. The presented values were confirmed by experiments with membranes with different carvacrol loadings ranging from 30% to 43% and release time of four hours. However, almost complete release of carvacrol from polyamide membranes is possible, which is illustrated by its release in air as presented in Figure A10 (Appendix A). Taking into account carvacrol’s high volatility, a kind of wet tissue packaging is recommended for storage of impregnated membranes.

### 3.5. Thoracic Cavity Model

**Figure 13:**
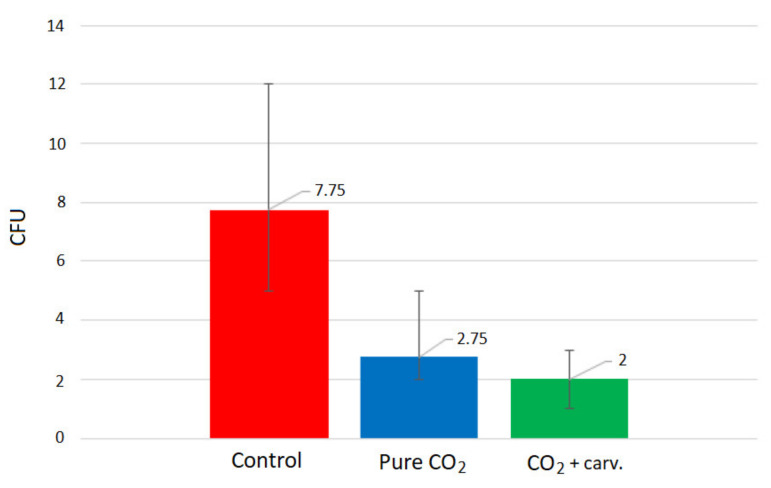
Contamination levels detected without CO_2_ insufflation (red), with insufflation using a neat polyamide membrane (blue), and insufflation using a carvacrol-loaded membrane (green).

An open thoracic cavity model, similar to the one applied by Persson and van der Linden [16], was used to compare contamination rates in case of insufflation using neat and carvacrol-loaded polyamide membranes. Experiments without any insufflation were performed as well for comparison. The open surgical wound model contained two 9 cm blood agar plates for detection of airborne bacteria contamination. The experimental setup is presented in Figure 1, and the procedure is described in detail in Materials and Methods (Section 2.2.6). Experiments were performed in five replicates, and each experiment lasted one hour. Membranes with carvacrol content between 30% and 34% were used for the study. The mean values of the contamination detected, represented by the number of colony-forming units (CFU), with maximal deviations from the mean are presented in Figure 13.

As can be seen, carbon dioxide insufflation decreased the contamination levels significantly. As a result, the CFU number decreased from an average value of 7.75 (without de-airing) to 2.75. This finding is consistent with the results of Persson and van der Linden [16] and Baumann and Cater [18], who showed that de-airing with carbon dioxide might prevent direct airborne contamination during cardiac surgery. Further experiments in our study demonstrated that using carvacrol-loaded membranes instead of neat polyamide membranes made it possible to decrease the contamination level by another 27% (decrease in the average CFU level from 2.75 to 2). Such a considerable decrease in contamination justifies the potential of these defined polymer structures loaded with antibacterial substance for application in carbon dioxide insufflation during open thoracic cavity surgeries.

## 4. Conclusions

Open thoracic cavity de-airing with pure carbon dioxide is used in open-heart surgery to prevent arterial embolism [14]. It has previously been shown that this procedure also prevents perioperative contamination of open surgical wounds [16,18]. Nevertheless, surgical infections are relatively common, with an incidence of approximately 5%. They are potentially fatal with a high percentage of complications in the form of sepsis, are difficult to treat, and incur high economic costs due to prolonged patient hospitalization and the use of very expensive antibiotics [17,42]. This study demonstrated that it was possible to decrease the contamination level in an open thoracic cavity model by another 27% compared to the standard de-airing procedure by employing filtration membranes loaded with carvacrol for carbon dioxide insufflation. Furthermore, the carvacrol-loaded membranes were produced in an environmentally friendly process using SSI with no waste generation. The process was characterized by fast impregnation in the first hour, whereby the suggested working pressure was 20 MPa. Polymer swelling was detectable in SEM and cross-filtration tests. However, membrane functionality was not considerably affected by this effect. The membranes with around 30–34 wt.% carvacrol were effective in the applied open thoracic cavity model. According to the results, membranes with carvacrol content of up to 43% retained functionality and may be considered for further evaluation for this application. Further studies are necessary to evaluate the durability and efficiency of membranes under different carbon dioxide flow rates and in different types of gas diffusers.

## Figures and Tables

**Table 1:** Wavenumbers and assignments (according to data from the literature) of bands obtained by FTIR analysis for carvacrol (C), pure polyamide (PA), and carvacrol-impregnated polyamide (PA + C).

Assignment	C	PA	PA + C	Ref.
C–H out-of-plane wagging	811 cm^−1^	-	810 cm^−1^	[29,30,31]
Carvacrol key characteristic peak	864 cm^−1^	-	867 cm^−1^	[29]
Carvacrol 1:2:4 substitution	994 cm^−1^	-	995 cm^−1^	[29,30,31]
Carvacrol ortho substitution	1115 cm^−1^	-	1118 cm^−1^	[29,30,31]
Carvacrol key characteristic peak	1173 cm^−1^	-	1178 cm^−1^	[29]
Carvacrol key characteristic peak	1249 cm^−1^		1257 cm^−1^	[29]
Aliphatic –CH_2_/–CH_3_ stretching	2958, 2926, and 2868 cm^−1^	-	Overtone with CH_2_ stretching vibration in PA	[29,30,31]
–OH stretching vibration	3378 cm^−1^	-	Overtone with strong N–H stretching vibration in PA	[29,30,31]
N–H stretching vibration	-	3299 and 3076 cm^−1^	3298 and 3065 cm^−1^	[32,33]
CH_2_ asymmetric stretching vibrations	-	2933 cm^−1^	2933 cm^−1^	[32,33,34]
CH_2_ symmetric stretching vibrations	-	2859 cm^−1^	2859 cm^−1^	[32,33,34]
C=O stretching of amide	-	1631 cm^−1^	1630 cm^−1^	[32,33,34]
N–H bending of amide	-	1536 cm^−1^	1534 cm^−1^	[32,33,34]
O=C–N group bending	-	689 cm^−1^	688 cm^−1^	[34]

## Data Availability

Samples are available upon request to the corresponding author.

## Sample Availability

Samples are available from the corresponding author upon request.

## References

[1] Weidner E. (2018). Impregnation via supercritical CO_2_—What we know and what we need to know. J. Supercrit. Fluids.

[2] Kiellow A.W., Henriksen O. (2009). Supercritical wood impregnation. J. Supercrit. Fluids.

[3] Iversen S.B., Larsen T., Henriksen O., Felsvang K. (2003). The world’s first commercial supercritical wood treatement plant. The International Society for the Advancement of Supercritical Fluids, Proceedings of the 6th International Symposium on Supercritical Fluids, Versailles, France, 28−30 April 2003.

[4] Van der Kraan M., Cid M.V.F., Woerlee G.F., Veugelers W.J.T., Witkamp G.J. (2007). Dyeing of natural and synthetic textiles in supercritical carbon dioxide with disperse reactive dyes. J. Supercrit. Fluids.

[5] Dyecoo. http://www.dyecoo.com/.

[6] Wolf C., Maninger J., Lederer K., Fruhwirth-Smounig H., Gamse T., Marr R. (2006). Stabilisation of crosslinked ultra-high molecular weight polyethylene (UHMW-PE)-acetabular components with α-tocopherol. J. Mater. Sci. Mater. Med..

[7] Thomas G., Rolf M., Christian W., Klaus L. (2007). Supercritical CO_2_ impregnation of polyethylene components for medical purposes. Hem. Ind..

[8] Costa V.P., Braga M.E., Guerra J.P., Duarte A.R., Duarte C.M., Leite E.O., Gil M.H., de Sousa H.C. (2010). Development of therapeutic contact lenses using a supercritical solvent impregnation method. J. Supercrit. Fluids.

[9] Costa V.P., Braga M.E., Duarte C.M., Alvarez-Lorenzo C., Concheiro A., Gil M.H., de Sousa H.C. (2010). Anti-glaucoma drug-loaded contact lenses prepared using supercritical solvent impregnation. J. Supercrit. Fluids.

[10] Champeau M., Thomassin J.-M., Tassaing T., Jérôme C. (2015). Drug loading of polymer implants by supercritical CO_2_ assisted impregnation: A review. J. Control. Release.

[11] Rojas A., Torres A., Galotto M.J., Guarda A., Romero J. (2020). Supercritical impregnation for food applications: A review of the effect of the operational variables on the active compound loading. Crit. Rev. Food Sci..

[12] Zizovic I., Trusek A., Tyrka M., Moric I., Senerovic I. (2021). Functionalization of polyamide microfiltration membranes by supercritical solvent impregnation. J. Supercrit. Fluids.

[13] Zizovic I., Tyrka M., Matya K., Moric I., Senerovic L., Trusek A. (2021). Functional modification of cellulose acetate microfiltration membranes by supercritical solvent impregnation. Molecules.

[14] Nyman J., Svenarud P., van der Linden J. (2019). Carbon dioxide de-airing in minimal invasive cardiac surgery, a new effective device. J. Cardiothorac. Surg..

[15] Orihashi K., Ueda T. (2019). “De-airing” in open heart surgery: Report from the CVSAP nation-wide survey and literature review. Gen. Thorac. Cardiovasc. Surg..

[16] Persson M., van der Linden J. (2004). Wound ventilation with carbon dioxide: A simple method to prevent direct airborne contamination during cardiac surgery?. J. Hosp. Infect..

[17] Persson M., Flock J., van der Linden J. (2003). Antiseptic wound ventilation with a gas diffuser: A new intraoperative method to prevent surgical wound infection?. J. Hosp. Infect..

[18] Baumann M., Cater J.E. (2018). The effect of heated CO_2_ insufflation in minimising surgical wound contamination during open surgery. Ann. Biomed. Eng..

[19] FDA, CFR—Code of Federal Regulations Title 21. https://www.accessdata.fda.gov/scripts/cdrh/cfdocs/cfcfr/CFRSearch.cfm?fr=172.515&SearchTerm=carvacrol.

[20] Nostro A., Blanco A.R.M., Cannatelli A., Enea V., Flamini G., Morelli I., Roccaro A.S., Alonzo V. (2004). Susceptibility of methicillin-resistant staphylococci to oregano essential oil, carvacrol and thymol. FEMS Microbiol. Lett..

[21] Leeke G.A., Santos R., King M.B. (2001). Vapor-liquid equilibria for the carbon dioxide + carvacrol system at elevated pressures. J. Chem. Eng. Data.

[22] Milovanovic S., Radetic M., Misic D., Asanin J., Leontijevic V., Ivanovic J., Zizovic I., Gordon S., Abidi N. (2017). High pressure modified cotton in wound dressing applications. Cotton Fibers: Characteristics, Uses and Performance.

[23] Milovanovic S., Adamovic T., Aksentijevic K., Misic D., Ivanovic J., Zizovic I. (2017). Cellulose acetate based material with antibacterial properties created by supercritical solvent impregnation. Int. J. Polym. Sci..

[24] Adamovic T., Milovanovic S., Markovic D., Zizovic I. (2018). Impregnation of cellulose acetate films with carvacrol using supercritical carbon dioxide. Tehnika.

[25] Dubois J., Grau E., Tassaing T., Dumon M. (2018). On the CO_2_ sorption and swelling of elastomers by supercritical CO_2_ as studied by in situ high pressure FTIR microscopy. J. Supercrt. Fluids.

[26] Bonavoglia B., Storti G., Morbidelli M., Rajendran A., Mazzotti M. (2006). Sorption and swelling of semicrystalline polymers.in supercritical CO_2_. J. Polym. Sci. Part B Polym. Phys..

[27] Houben M., van Geijn R., Essen M., Borneman Z., Nijmeije K. (2021). Supercritical CO_2_ permeation in glassy polyimide membranes. J. Memb. Sci..

[28] Marković D., Milovanović S., De Clerck K., Zizovic I., Stojanović D., Radetić M. (2018). Development of material with strong antimicrobial activity by high pressure CO_2_ impregnation of polyamide nanofibers with thymol. J. CO_2_ Util..

[29] Bertuola M., Fagali N., de Mele F.L. (2020). Detection of carvacrol in essential oils by electrochemical polymerization. Heliyon.

[30] Valderrama A.S.S., De Rojas G.C. (2017). Traceability of active compounds of essential oils in antimicrobial food packaging using a chemometric method by ATR-FTIR. Am. J. Analyt. Chem..

[31] Schulz H., Quilitzsch R., Krüger H. (2003). Rapid evaluation and quantitative analysis of thyme, oregano and chamomile essential oils by ATR-IR and NIR spectroscopy. J. Mol. Struct..

[32] Lopes E.S., Domingos E., Neves R.S., Romão W., Sousa K.R., Valaski R., Archanjo B.S., Souza F.G., Silva A.M., Kuznetsov A. (2016). The role of intermolecular interactions in polyaniline/polyamide-6,6 pressure-sensitive blends studied by DFT and ^1^H NMR. Eur. Polym. J..

[33] Pavliňák D., Hnilica J., Quade A., Schäfer J., Alberti M., Kudrle V. (2014). Functionalisation and pore size control of electrospun PA6 nanofibres using a microwave jet plasma. Polym. Degrad. Stabil..

[34] Kiakhani M.S., Safapour S. (2016). Improvement of dyeing and antimicrobial properties of nylon fabrics modified using chitosan-poly(propylene imine) dendrimer hybrid. J. Ind. Eng. Chem..

[35] Acero E.H., Ribitsch D., Rodriguez R.D., Dellacher A., Zitzenbacher S., Marold A., Greimel K.J., Schroeder M., Kandelbauer A., Heumann S. (2012). Two-step enzymatic function of polyamide with phenolics. J. Mol. Catal..

[36] Kaziran S.G., Martirosyan G.G. (2002). Spectroscopy of polymer/drug formulations processed with supercritical fluids: In situ ATR–IR and Raman study of impregnation of ibuprofen into PVP. Int. J. Pharm..

[37] Arnaudov M.G., Ivanova B.B., Dinkov S.G. (2004). A reducing-difference IR-spectral study of 4-aminopyridine. Cent. Eur. J. Chem..

[38] Good Vibrations with IR Spectroscopy. http://www1.udel.edu/chem/fox/IR_lectureNotes.pdf.

[39] Espinosa-Acosta G., Ramos-Jacques A.L., Molina G.A., Maya-Cornejo J., Esparza R., Hernandez-Martinez A.R., Sánchez-González I., Estevez M. (2018). Stability analysis of anthocyanins using alcoholic extracts from black carrot (*Daucus carota* ssp. Sativus Var. *Atrorubens* Alef.). Molecules.

[40] Milovanovic S., Markovic D., Aksentijevic K., Stojanovic D.B., Ivanovic J., Zizovic I. (2016). Application of cellulose acetate for controlled release of thymol. Carbohyd. Polym..

[41] Milovanovic S., Stamenic M., Markovic D., Ivanovic J., Zizovic I. (2015). Supercritical impregnation of cellulose acetate with thymol. J. Supercrit. Fluids.

[42] Gottrup F. (2000). Prevention of surgical-wound infections. N. Engl. J. Med..

